# Prognostic gene signature profiles of hepatitis C-related early-stage liver cirrhosis

**DOI:** 10.1016/j.gdata.2014.10.007

**Published:** 2014-10-12

**Authors:** Anu Venkatesh, Xiaochen Sun, Yujin Hoshida

**Affiliations:** Liver Cancer Program, Tisch Cancer Institute, Division of Liver Diseases, Department of Medicine, Icahn School of Medicine at Mount Sinai, New York, NY, USA

**Keywords:** Hepatitis C, Cirrhosis, Gene expression, NanoString nCounter, Prognostic

## Abstract

The rate of hepatitis C virus (HCV) related liver cirrhosis and subsequent cancer development is increasing and raising the risk of related mortality and morbidity. To address this issue, we aimed to develop a prognostic index that can be used to stratify patients for risk of disease progression. This index was developed in part by using a gene signature test implemented in a clinically applicable digital transcript counting platform (NanoString nCounter system). A cohort of 145 U.S. patients with HCV-related early-stage cirrhosis was analyzed by using the assay. This dataset (GEO accession number GPL17230) provides information of expression levels of the prognostic genes in the cohort.

**Table:** 

Specification	
Organism/cell line/tissue	*Homo Sapiens, tissue*
Sex	*Male and Female*
Sequencer or array type	*NanoString nCounter assay*
Data format	*Raw data: .txt, Normalized data: SOFT, MINiML, Series Matrix* *(.txt)*
Experimental factors	*Gene expression in tissue*
Experimental features	*Gene expression profiles were obtained by using total RNA extracted from formalin-fixed paraffin-embedded liver biopsy tissue specimens.*
Consent	*Requirement of consent was waived on condition that all patients be made anonymous*
Sample source location	*Mass General Hospital, Boston MA, U.S., 42.3628° N, 71.0686° W*

## Direct link to deposited data

Deposited data can be found here: http://www.ncbi.nlm.nih.gov/geo/query/acc.cgi?acc=GSE54100.

## Experimental design, materials and methods

### Tissue samples

All of the patient samples were collected from liver biopsies performed at Massachusetts General Hospital. All patients had a histological diagnosis of cirrhosis and had confirmed HCV infection based on positivity of serum HCV antibody or RNA.

### RNA preparation

Total RNA was prepared from 10 μm-thick formalin-fixed paraffin-embedded (FFPE) tissue sections by using a High Pure RNA Paraffin kit (Roche) according to the manufacturer's instructions. RNA quality was assessed by qRT-PCR of a housekeeping gene RPL13A as previously described [1]. All samples were confirmed to have a crossover threshold (Ct) value less than 33 and an RNA concentration greater than 20 ng/μl, and 100 to 500 ng total RNA was subjected to the expression profiling.

### NanoString nCounter assay and data normalization

The 186-gene signature defined and validated in our previous studies [1], [2] was implemented in the nCounter digital transcript counting assay specifically designed for the analysis of clinical specimen including archived FFPE tissue (GEO accession number GPL17230) [3], [4], [5], [6], [7]. The assay was run according to the manufacturer's instruction on a high sensitivity “green” New Prep Plate, and hybridized cartridges were scanned using an nCounter Digital Analyzer. Raw transcript count data were log-transformed (base 2) and scaled by geometric mean of control probe data (annotated as “normalization_gene”) by using the NanoString normalizer module implemented in the GenePattern genomic analysis toolkit (www.broadinstitute.org/genepattern). A floor value of 1.1 was applied before the log-transformation.

### Bioinformatics data analysis

The 186-gene signature-based outcome prediction was performed using the nearest template prediction algorithm implemented in the GenePattern Nearest Template Prediction module [8] and the prediction model reported in our initial publication without making any modification [1]. The prognostic association of the gene signature-based prediction was evaluated by the Kaplan–Meier curves, log-rank test, and Cox regression modeling.

## Financial support

This research was supported by the National Institutes of Health (DK099558 to Y.H.; DA033541, DK098079).

## Conflict of interest

Y.H. is one of the three investigators on a pending patent application entitled “Compositions, kits, and methods for detecting, characterizing, preventing, and treating hepatic disorders (USPTO application #: #20110263441)”. NanoString, Inc. has secured the option to an exclusive worldwide license. NanoString has no role in the conduction of the current study. There is no other relevant declaration relating to employment, consultancy, patents, products in development or modified products etc.
